# A Large Language Model Screening Tool to Target Patients for Best Practice Alerts: Development and Validation

**DOI:** 10.2196/49886

**Published:** 2023-11-27

**Authors:** Thomas Savage, John Wang, Lisa Shieh

**Affiliations:** 1 Division of Hospital Medicine Department of Medicine Stanford University Palo Alto, CA United States; 2 Divison of Gastroenterology and Hepatology Department of Medicine Stanford University Palo Alto, CA United States

**Keywords:** large language models, language models, language model, EHR, health record, health records, quality improvement, Artificial Intelligence, Natural Language Processing

## Abstract

**Background:**

Best Practice Alerts (BPAs) are alert messages to physicians in the electronic health record that are used to encourage appropriate use of health care resources. While these alerts are helpful in both improving care and reducing costs, BPAs are often broadly applied nonselectively across entire patient populations. The development of large language models (LLMs) provides an opportunity to selectively identify patients for BPAs.

**Objective:**

In this paper, we present an example case where an LLM screening tool is used to select patients appropriate for a BPA encouraging the prescription of deep vein thrombosis (DVT) anticoagulation prophylaxis. The artificial intelligence (AI) screening tool was developed to identify patients experiencing acute bleeding and exclude them from receiving a DVT prophylaxis BPA.

**Methods:**

Our AI screening tool used a BioMed-RoBERTa (Robustly Optimized Bidirectional Encoder Representations from Transformers Pretraining Approach; AllenAI) model to perform classification of physician notes, identifying patients without active bleeding and thus appropriate for a thromboembolism prophylaxis BPA. The BioMed-RoBERTa model was fine-tuned using 500 history and physical notes of patients from the MIMIC-III (Medical Information Mart for Intensive Care) database who were not prescribed anticoagulation. A development set of 300 MIMIC patient notes was used to determine the model’s hyperparameters, and a separate test set of 300 patient notes was used to evaluate the screening tool.

**Results:**

Our MIMIC-III test set population of 300 patients included 72 patients with bleeding (ie, were not appropriate for a DVT prophylaxis BPA) and 228 without bleeding who were appropriate for a DVT prophylaxis BPA. The AI screening tool achieved impressive accuracy with a precision-recall area under the curve of 0.82 (95% CI 0.75-0.89) and a receiver operator curve area under the curve of 0.89 (95% CI 0.84-0.94). The screening tool reduced the number of patients who would trigger an alert by 20% (240 instead of 300 alerts) and increased alert applicability by 14.8% (218 [90.8%] positive alerts from 240 total alerts instead of 228 [76%] positive alerts from 300 total alerts), compared to nonselectively sending alerts for all patients.

**Conclusions:**

These results show a proof of concept on how language models can be used as a screening tool for BPAs. We provide an example AI screening tool that uses a HIPAA (Health Insurance Portability and Accountability Act)–compliant BioMed-RoBERTa model deployed with minimal computing power. Larger models (eg, Generative Pre-trained Transformers–3, Generative Pre-trained Transformers–4, and Pathways Language Model) will exhibit superior performance but require data use agreements to be HIPAA compliant. We anticipate LLMs to revolutionize quality improvement in hospital medicine.

## Introduction

Large language models (LLMs) are an exciting development in the field of natural language processing and present tremendous potential for application to clinical medicine. Language models such as Bidirectional Encoder Representations from Transformers (BERT), Robustly Optimized BERT Pretraining Approach (RoBERTa), and Generative Pre-trained Transformers (GPT) can perform complex tasks such as text classification, question answering, and text generation, which have the potential to augment physicians in providing care for patients. A clinical application of significant potential is LLM optimization of quality improvement electronic health record (EHR) Best Practice Alerts (BPAs).

EHR BPAs are powerful tools to improve patient care outcomes. Their widespread use has demonstrated better adherence to clinical guidelines, fewer medication errors, improved diabetes management, more appropriate antimicrobial prescribing, and higher rates of ambulatory preventive care, among others [1]. Nevertheless, despite these benefits, the overuse of BPAs can cause alarm fatigue and desensitization that can even lead to patient harm, as described by the Joint Commission Alert System Safety Report of 2014 [2,3]. Current BPAs are often overconservative, broadly applied to all possible patients, resulting in up to 49% to 96% of alerts being overridden or ignored [4]. A BPA will frequently be obviously inappropriate or not applicable to the patient, resulting in the physician becoming desensitized to the alert and more likely to ignore a valid alert in the future [5,6]. LLMs offer an opportunity to screen appropriate patients for BPAs.

In this paper, we propose how language models can be leveraged to read a physician’s note and screen whether a patient is appropriate for a BPA. We examine an example case using a BioMed-RoBERTa artificial intelligence (AI) screening tool that selectively identifies patients who are appropriate for a deep vein thrombosis prophylaxis. Our screening tool reads each history and physical note to identify whether a patient has active bleeding (a contraindication to anticoagulation) and excludes those patients with bleeding from receiving a deep vein thrombosis prophylaxis BPA. We hope our methods will encourage the use of language models to screen patients for BPAs and improve EHR workflow.

## Methods

### Patient Note Data Set

The MIMIC-III (Medical Information Mart for Intensive Care) data set is publicly available and consists of more than 60,000 intensive care unit admissions from the Brigham and Women’s Hospital system [7-9]. The data set includes EHR-equivalent patient data, including physician notes and medication administration data.

For this study, we selected history and physical notes from MIMIC-III for patients who did not receive an anticoagulant (therapeutic or prophylactic) at the time of admission. We selected the first 1100 history and physical notes in chronological time stamp order for inclusion in this study. Each note described a unique patient, meaning no 2 notes had the same patient identifier (subject_id). The subject_id and time stamp for each note included in our study were recorded and can be shared upon request with proper registry for the MIMIC database on the PhysioNet platform. Due to LLM token limits, patient notes were truncated to 2000 characters (RoBERTa models can receive a maximum of 512 tokens, which translates to roughly 2000 characters).

The MIMIC-III notes were then split into a training set (first 500 notes), development set (middle 300 notes), and test set (final 300 notes). The training set was used to fine-tune the AI model, the development set was used to determine model hyperparameters (learning rate, batch number, training epochs, and seed number), and the test set was used for screening tool evaluation. Cohort information can be shared upon request with registry for the MIMIC database on the PhysioNet platform.

In total, 2 physicians (TS and JW) reviewed all notes and labeled each note as either describing a patient with active bleeding or without active bleeding. Active bleeding was defined as the loss of blood from the vessels of the body either by documented visual or imaging evidence of bleeding as well as suspected bleeding documented by the physician author. These labels were used as the gold standard labels for model evaluation. If there was disagreement between labels assigned by the 2 reviewers, the case was discussed to reach a final label designation.

### Language Model

The language model used in this screening tool was BioMed-RoBERTa [10] by AllenAI. BioMed-RoBERTa is a bidirectional transformer encoder based on the RoBERTa-base model published by Liu et al [11]. BioMed-RoBERTa continued pretraining beyond RoBERTa-base using 2.68 million scientific papers in the domains of biology and medicine from the Semantic Scholar corpus [10]. Therefore BioMed-RoBERTa has increased proficiency in the subjects of biology and medicine compared to RoBERTa-base.

The optimal hyperparameter settings were identified as 9 training epochs, a training batch size of 2, a learning rate of 4 × 10^–5^, and a starting seed of 9. The full code can be referenced in Multimedia Appendix 1

The model performed classification for each history and physical note, classifying the note as either describing active bleeding or no active bleeding.

### Code and Computing Environment

Model training and evaluation were completed in a PyCharm notebook using an Apple M2 GPU. Full code can be referenced in Multimedia Appendix 1.

### Statistical Methods

Model classification performance was evaluated on a test set of 300 MIMIC patient notes. Classification performance was evaluated by a precision-recall area under the curve (AUC), a receiver operating characteristic (ROC) AUC, sensitivity, and specificity. Error was calculated using a replacement bootstraps method with 4000 bootstrapped populations from the test set. The full code can be referenced in Multimedia Appendix 1.

The statistic “increase in alert applicability” was calculated from equation 1 below.



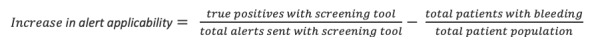



### Ethical Considerations

The MIMIC patient records database is available as an open repository for credential users registered within the PhysioNet platform. The patient records within the MIMIC database have been deidentified, and patient identifiers have been removed according to the HIPAA (Health Insurance Portability and Accountability Act) Safe Harbor provision. This study was approved and authorized by the PhysioNet Team.

The collection of patient information and creation of the MIMIC research resource was reviewed by the institutional review board at the Beth Israel Deaconess Medical Center (protocol ID 73104), who granted a waiver of informed consent and approved the data sharing initiative. Due to the waiver of informed consent and anonymous nature of the MIMIC data set, patients were not compensated for inclusion in the database or this research.

## Results

**Figure 1:**
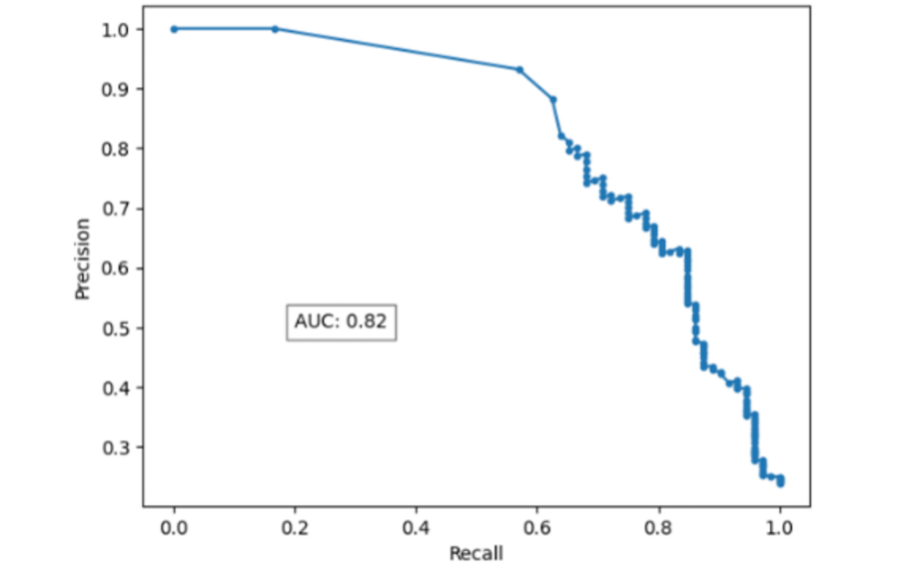
Precision-recall curve for the BioMed-RoBERTa model. AUC: area under the curve; RoBERTa: Robustly Optimized Bidirectional Encoder Representations from Transformers Pretraining Approach.

**Figure 2:**
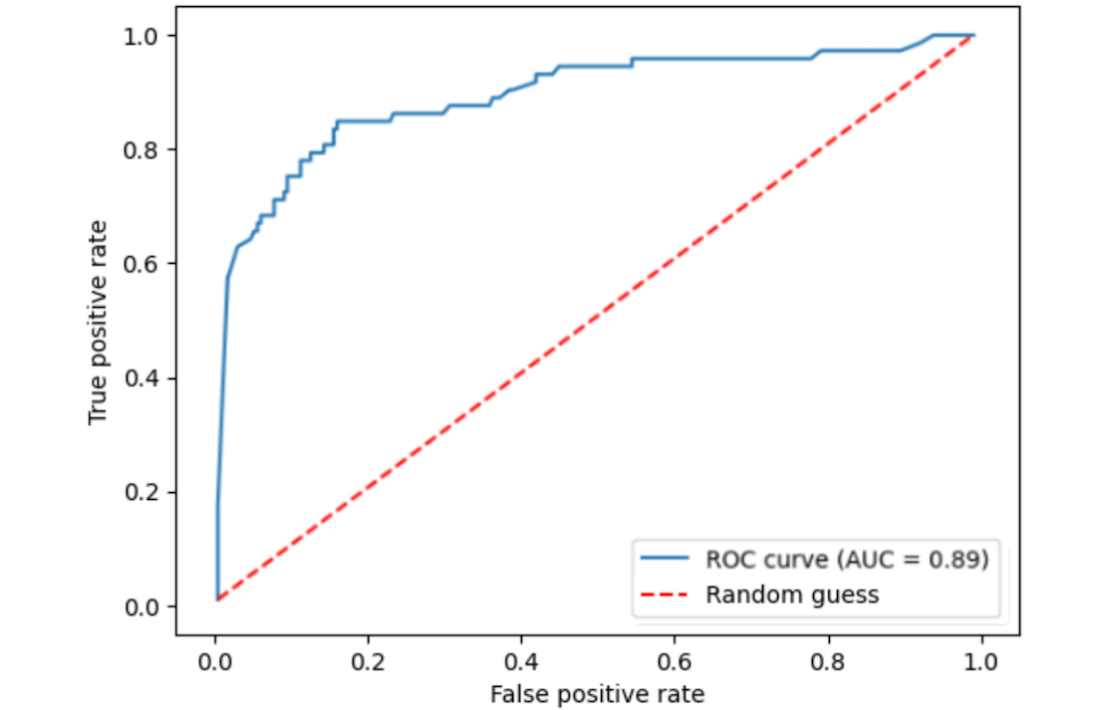
ROC curve for the BioMed-RoBERTa model. AUC: area under the curve; ROC: receiver operating characteristic; RoBERTa: Robustly Optimized Bidirectional Encoder Representations from Transformers Pretraining Approach.

The test set of 300 MIMIC patient notes included 72 patients experiencing acute bleeding and 228 patients not experiencing acute bleeding. The AI screening tool was able to achieve a precision-recall curve AUC of 0.82 (95% CI 0.75-0.89; Figure 1) and a ROC AUC of 0.89 (95% CI 0.84-0.94; Figure 2). Sensitivity was found to be 67% (95% CI 55%-77%) and specificity was found to be 95% (95% CI 92%-97%). Sensitivity, specificity, and confusion matrix data can be found in Table 1.

If this model were used to identify patients for a thromboembolism prophylaxis BPA in place of a nonselective strategy deploying an alert for all patients, this model would reduce the number of BPA alerts by 20% (240 alerts sent instead of 300) and increase applicability of the BPA by 14.8% (equation 1). This model misclassified 12 patients who did not have bleeding and would be appropriate to receive a thromboembolism prophylaxis BPA. This model captured 94.7% (216/228) of the population who would be appropriate for an alert. The model achieved a positive predictive value of 80% and a negative predictive value of 90%. Full results can be found in Table 1.

A review of notes of patients who were incorrectly classified found trends where the tool underperformed. Of the 24 patients misclassified as without bleeding, the model had difficulty identifying bleeding if it was a secondary complaint (6 patients), meaning if the patient primarily presented for another chief complaint and bleeding was incidentally noted. The model also had difficulty interpreting atypical or rare abbreviations that denoted bleeding (6 patients). This included recognizing “subdural” as an abbreviation for subdural hemorrhage (2 patients), “EBL” as an abbreviation for estimated blood loss (2 patients), and “SAH” as an abbreviation for subarachnoid hemorrhage (2 patients). For the 12 patients misclassified as with bleeding, the model had difficulty with negated bleeding phrases (7 patients), for example “denies melena,” as well as notes describing chronic anemia (2 patients) or previous bleeding events in the distant past that were not currently active (2 patients).

**Table 1:** Confusion matrix for the BioMed-RoBERTa^a^ model’s classification results.

LLM^b^ classification	Physician label		
	Positive	Negative	Total
Positive	48	12	60
Negative	24	216	240
Total	72	228	300

^a^RoBERTa: Robustly Optimized Bidirectional Encoder Representations from Transformers Pretraining Approach.

^b^LLM: large language model.

## Discussion

### Principal Findings

This prototype AI screening tool shows how language models can be leveraged to optimize EHR BPAs. Our example screening tool reduced the number of patients who would trigger an alert by 20% while also increasing applicability of alerts by 14.8% compared to nonselectively sending alerts for all patients. The tool achieved impressive accuracy with a precision-recall AUC of 0.82 and ROC AUC of 0.89. We demonstrate how the ability for language models to interpret physician notes to classify patients offers a new method of targeting the most appropriate patients for a BPA with high accuracy.

Language model screening tools to target patients for BPAs are most appropriate when the alert’s goal is to catch most target patients but the consequence of missing a few patients is not significant. As demonstrated by our example, language models are accurate but do occasionally misclassify patients. Example use cases appropriate for an AI screening tool would be identifying patients for discontinuation of telemetry monitoring, ascribing correct level of nursing care (inpatient vs observation), and encouraging best prescribing practices for blood products. Inappropriate examples would be situations such as medication interactions or isolation precautions. In those cases, a nonselective blanket rule-based BPA would be more appropriate.

The limitations of the screening tool evaluated in this study were its relatively small training set of 500 patient notes as well as the use of the BioMed-RoBERTa model rather than a larger model such as GPT-3 or GPT-4. Increasing the size of our training set would likely decrease many of the misclassification errors seen by our screening tool. Specifically, the misclassification errors due to misinterpretation of atypical or rare abbreviations for bleeding would be reduced with an increased training set size. A larger base model would also reduce many of the errors observed by our screening tool. RoBERTa models contain 110 million parameters trained on 160 GB of text data [11]. Larger models, such as GPT-3, consist of 175 billion parameters trained on over 40 TB of text data, nearly 10 times the size of RoBERTa, and demonstrate superior performance in negation detection and semantic understanding [12,13]. Therefore, a larger model would reduce the misclassification errors caused by misinterpretation of negated bleeding or previous bleeding events in the distant past. Our study chose BioMed-RoBERTa because its smaller size allows it to be trained on a local computing environment compliant with the HIPAA 1996 data privacy standards. Future investigations will need to secure the necessary data use agreements to use larger models (eg, GPT-3, GPT-4, or Pathways Language Model) with medical grade data, where we anticipate screening tool performance will be significantly improved.

### Conclusions

In this paper, we proposed a new application for LLMs in medicine. Quality improvement BPAs can leverage LLMs to read physician notes and better identify patients for BPA alerts. We provide an example case which demonstrates the ability of a BioMed-RoBERTa to achieve impressive classification accuracy. We anticipate that as the field of clinical natural language processing continues to grow, with increasing access to larger language models, LLMs will revolutionize the field of clinical quality improvement.

## Appendix

Multimedia Appendix 1 Python code for training and evaluating the large language model (Robustly Optimized Bidirectional Encoder Representations from Transformers Pretraining Approach; RoBERTa) screening tool used in this investigation.

## Abbreviations

AI: artificial intelligence
AUC: area under the curve
BERT: Bidirectional Encoder Representations from Transformers
BPA: Best Practice Alert
DVT: deep vein thrombosis
EHR: electronic health record
GPT: Generative Pre-trained Transformers
HIPAA: Health Insurance Portability and Accountability Act
LLM: large language model
MIMIC: Medical Information Mart for Intensive Care
RoBERTa: Robustly Optimized Bidirectional Encoder Representations from Transformers Pretraining Approach
ROC: receiver operating characteristic

## Data Availability

The Python code used for this investigation is available in Multimedia Appendix 1. MIMIC (Medical Information Mart for Intensive Care) patient data is an open repository requiring registration with the PhysioNet platform. Cohort information of the history and physical notes used to train, develop, and test our screening tool in can be shared upon request with proper registry for the MIMIC database on the PhysioNet platform.
