# Supplementary material for: A Large Language Model Screening Tool to Target Patients for Best Practice Alerts: Development and Validation
Source: JMIR Med Inform. 2023 Nov 27;11:e49886. doi: 10.2196/49886 (PMC10714262; doi:10.2196/49886)
Supplement: Multimedia Appendix 1 [file medinform_v11i1e49886_app1.pdf]

```
### Modules to Install
# pip3 install simpletransformers "transformers==4.30.2"
# pip3 install simpletransformers -q
# pip3 install scikit-multilearn -q
# pip3 install scikit-plot
# pip3 install pandas
# pip3 install numpy
# pip3 install matplotlib
######
import pandas as pd
from skmultilearn.model_selection import iterative_train_test_split
from simpletransformers.classification import (MultiLabelClassificationModel,
MultiLabelClassificationArgs)
import logging
from simpletransformers.classification import ClassificationModel
import matplotlib.pyplot as plt
from sklearn.metrics import roc_curve, auc
from sklearn.metrics import confusion_matrix, classification_report
from sklearn.metrics import precision_recall_curve, auc
from sklearn.model_selection import train_test_split
from sklearn.linear_model import LogisticRegression
####
"""Downloading a csv file that contains training MIMIC III notes to train the RoBERTa Model.
The below code also formats the file for input into the RoBERTa model."""

data = pd.read_csv("training.csv", engine='python', error_bad_lines=False)
data = data.sample(frac=1, random_state=1)
x = data['text'].values
y = data['label'].values
df1 = pd.DataFrame(x)
df2 = pd.DataFrame(y)
train_data = pd.concat([df1, df2], axis=1)
train_data.columns = ['text', 'labels']

"""Downloading a csv file that contains test MIMIC III notes to evaluate the RoBERTa Model.
The below code also formats the file for input into the RoBERTa model."""

df = pd.read_csv("test.csv", engine='python', error_bad_lines=False)
df.columns = ["text", "labels"]
df['labels'] = df['labels'].astype(int)

"""**Model Specifications**

- max_seq_length: 512
```

- train_batch_size: 2
- num_train_epochs: 9
- Manual_seed: 9
- Learning Rate: Default

```
"""

# Model configuration
model_args = MultiLabelClassificationArgs(
num_train_epochs=9,
max_seq_length= 512,
train_batch_size=2,
overwrite_output_dir= True,
manual_seed = 9,
no_cache=True,
use_multiprocessing_for_evaluation=False,
use_multiprocessing=False
)
# Create the classification model
model = MultiLabelClassificationModel(
  "roberta",
  "allenai/biomed_roberta_base",
  num_labels=1,
  use_cuda=False, args=model_args)


# Train the model using the training set data
model.train_model(train_data, use_cuda=False)


#Make predictions with the model using the test data
predictions, raw_outputs = model.predict(df['text'].tolist())
labels = df['labels'].tolist()  # Physician labels for the input texts
true_labels = labels

# Compute the ROC curve
fpr, tpr, thresholds = roc_curve(true_labels, raw_outputs)

# Calculate the area under the ROC curve (AUC)
roc_auc = auc(fpr, tpr)
print(roc_auc)
# Plot the ROC curve
plt.figure()
```

```
plt.plot(fpr, tpr, label='ROC curve (AUC = {:.2f})'.format(roc_auc))
plt.plot([0, 1], [0, 1], linestyle='--', color='r', label='Random Guess')
plt.xlabel('False Positive Rate')
plt.ylabel('True Positive Rate')
plt.title('Receiver Operating Characteristic')
plt.legend(loc='lower right')
plt.show()

# Calculating Sensitivity and Specificity
# Displaying elements of the confusion matrix
threshold = [0.5]
predicted_labels = [1 if value > threshold else 0 for value in raw_outputs]
cm = confusion_matrix(true_labels, predicted_labels)
tn, fp, fn, tp = cm.ravel()

sensitivity = tp / (tp + fn)

specificity = tn / (tn + fp)

print("TP: {:.2f}".format(tp))
print("FP: {:.2f}".format(fp))
print("TN: {:.2f}".format(tn))
print("FN: {:.2f}".format(fn))

print("Sensitivity: {:.2f}".format(sensitivity))
print("Specificity: {:.2f}".format(specificity))
print("\nClassification Report:")
print(classification_report(true_labels, predicted_labels))

# Calculating a Precision-Recall Curve with AUC
precision, recall, _ = precision_recall_curve(labels, raw_outputs)
auc_score = auc(recall, precision)
print('AUC: %.3f' % auc_score)

plt.plot(recall, precision, marker='.', label='Logistic')
plt.xlabel('Recall')
plt.ylabel('Precision')
plt.text(0.2, 0.5, f'AUC: {auc_score:.2f}', bbox=dict(facecolor='white', alpha=0.5))
plt.title('Precision-Recall curve')
plt.show()

# Save model results to a text file
import csv
with open('../../Downloads/mimic_run', 'w', newline='') as file:
```

```
  writer = csv.writer(file)
  writer.writerows(raw_outputs)

"""Calculation of error via bootstraps method for:
- Precision-Recall AUC
- Sensitivity
- Specificity
"""

from sklearn.metrics import precision_recall_curve, auc
import numpy as np

### computing PRC AUC error with bootstraps
def compute_bootstrap_auc_ci(y_true, y_pred, n_bootstraps=4000, confidence_level=0.95):
    n = len(y_true)
    assert len(y_pred) == n

    auc_values = []
    for _ in range(n_bootstraps):
        # Create a random index for bootstrapping, with replacement
        indices = np.random.choice(range(n), size=n, replace=True)

        # Generate the sample set and calculate precision, recall, and AUC
        sample_true = y_true[indices]
        sample_pred = y_pred[indices]
        precision, recall, _ = precision_recall_curve(sample_true, sample_pred)
        auc_val = auc(recall, precision)
        auc_values.append(auc_val)

    # Compute the lower and upper percentiles
    lower = np.percentile(auc_values, (1-confidence_level)/2 * 100)
    upper = np.percentile(auc_values, (1+confidence_level)/2 * 100)

    return (lower, upper)

array = np.array(df['labels'])

compute_bootstrap_auc_ci(y_true = array, y_pred=raw_outputs, n_bootstraps=4000,
confidence_level=0.95)

from sklearn.metrics import roc_curve, roc_auc_score


### computing ROC AUC error with bootstraps
```

```
def compute_bootstrap_auc_ci2(y_true, y_pred, n_bootstraps=4000, confidence_level=0.95):
    n = len(y_true)
    assert len(y_pred) == n

    auc_values = []
    for _ in range(n_bootstraps):
        # Create a random index for bootstrapping, with replacement
        indices = np.random.choice(range(n), size=n, replace=True)

        # Generate the sample set and calculate FPR, TPR, and AUC
        sample_true = y_true[indices]
        sample_pred = y_pred[indices]
        fpr, tpr, _ = roc_curve(sample_true, sample_pred)
        auc_val = roc_auc_score(sample_true, sample_pred)
        auc_values.append(auc_val)

    # Compute the lower and upper percentiles
    lower = np.percentile(auc_values, (1-confidence_level)/2 * 100)
    upper = np.percentile(auc_values, (1+confidence_level)/2 * 100)

    return (lower, upper)

compute_bootstrap_auc_ci2(y_true = array, y_pred=raw_outputs, n_bootstraps=4000,
confidence_level=0.95)

from sklearn.metrics import confusion_matrix
import numpy as np

def compute_bootstrap_sens_spec_ci(y_true, y_pred, threshold=0.5, n_bootstraps=2000,
confidence_level=0.95):
    n = len(y_true)
    assert len(y_pred) == n

    # Initialize lists to store sensitivity and specificity values
    sensitivity_values = []
    specificity_values = []

    for _ in range(n_bootstraps):
        # Create a random index for bootstrapping, with replacement
        indices = np.random.choice(range(n), size=n, replace=True)

        # Generate the sample set and calculate sensitivity and specificity
        sample_true = y_true[indices]
        sample_pred = y_pred[indices] > threshold  # Apply threshold
```

```
        tn, fp, fn, tp = confusion_matrix(sample_true, sample_pred).ravel()

        sensitivity = tp / (tp + fn)
        specificity = tn / (tn + fp)

        sensitivity_values.append(sensitivity)
        specificity_values.append(specificity)

    # Compute the lower and upper percentiles for sensitivity and specificity
    sensitivity_lower = np.percentile(sensitivity_values, (1-confidence_level)/2 * 100)
    sensitivity_upper = np.percentile(sensitivity_values, (1+confidence_level)/2 * 100)

    specificity_lower = np.percentile(specificity_values, (1-confidence_level)/2 * 100)
    specificity_upper = np.percentile(specificity_values, (1+confidence_level)/2 * 100)

    return (sensitivity_lower, sensitivity_upper, specificity_lower, specificity_upper)

compute_bootstrap_sens_spec_ci(array,raw_outputs,0.5,4000,0.95)
```
